# Mid-upper arm circumference in pregnant women and birth weight in newborns as substitute for skinfold thickness: findings from the MAASTHI cohort study, India

**DOI:** 10.1186/s12884-021-03915-1

**Published:** 2021-07-06

**Authors:** Giridhara R. Babu, Aritra Das, Eunice Lobo, Deepa R, Daisy A. John, Prashanth Thankachan, Sonalini Khetrapal, Sara E. Benjamin-Neelon, GVS Murthy

**Affiliations:** 1 Indian Institute of Public Health-Bengaluru, Public Health Foundation of India (PHFI), Bengaluru, India; 2 Wellcome Trust-DBT India Alliance Intermediate Research Fellow in Public Health, Hyderabad, India; 3 Bihar Technical Support Program, CARE India, Patna, India; 4 Nutrition Division, St Johns Research Institute, Bengaluru, India; 5 Asian Development Bank (ADB) NCR - National Capital Region, Manila, Philippines; 6 Department of Health, Behavior and Society, Johns Hopkins Bloomberg School of Public Health, Baltimore, MD USA; 7 Indian Institute of Public Health-Hyderabad, Public Health Foundation of India (PHFI), Hyderabad, India; 8 Public Health Eye Care & Disability, London School of Hygiene & Tropical Medicine, London, United Kingdom

**Keywords:** anthropometric markers, India, Karnataka, skinfold thickness

## Abstract

**Background:**

Estimating total body fat in public hospitals using gold-standard measurements such as air displacement plethysmography (ADP), deuterium oxide dilution, or dual-energy X-ray absorptiometry (DXA) is unaffordable, and it is challenging to use skinfold thickness. We aimed to identify the appropriate substitute marker for skinfold thickness to estimate total body fat in pregnant women and infants.

**Methods:**

The study is part of a prospective cohort study titled MAASTHI in Bengaluru, from 2016 to 19. Anthropometric measurements such as body weight, head circumference, mid-upper arm circumference (MUAC), and skinfold thickness were measured in pregnant women between 14 and 36 weeks of gestational age; while measurements such as birth weight, head, chest, waist, hip, mid-upper arm circumference, and skinfold thickness were recorded for newborns. We calculated *Kappa* statistics to assess agreement between these anthropometric markers with skinfold thickness.

**Results:**

We found the highest amount of agreement between total skinfold thickness and MUAC (*Kappa* statistic, 0.42; 95 % CI 0.38–0.46) in pregnant women. For newborns, the highest agreement with total skinfold thickness was with birth weight (0.57; 95 % CI 0.52–0.60). Our results indicate that MUAC higher than 29.2 cm can serve as a suitable alternative to total skinfolds-based assessments for obesity screening in pregnancy in public facilities. Similarly, a birth weight cut-off of 3.45 kg can be considered for classifying obesity among newborns.

**Conclusion:**

Mid-upper arm circumference and birth weight can be used as markers of skinfold thickness, reflecting total body fat in pregnant women and the infant, respectively. These two anthropometric measurements could substitute for skinfold thickness in low- and middle-income urban India settings.

**Supplementary Information:**

The online version contains supplementary material available at 10.1186/s12884-021-03915-1.

## Introduction

The increasing prevalence of overweight and obesity among children is a significant public health issue attributing to immediate and long-term health problems [1]. However, the available estimates of obesity are highly variable in India, suggesting a range of 1 to 29 % of children [2–5] and 11.1 % in pregnant women [6]. There is an intergenerational cycle of perpetuating association of obesity in mothers with that of children, leading to a myriad of diseases such as type 2 diabetes mellitus (T2DM), dyslipidemia, and cardiovascular disease (CVD). Obesity has increased in adults and children owing to the epidemiological, demographical, and nutritional transition in India. From 1990 to 2017, the prevalence of children with obesity has increased annually by 4.98 %, with a projected prevalence of 17.5 % by 2030 [7]. In order to start effective strategies to reduce adverse outcomes, it is necessary to evaluate pregnant women and children for obesity using reliable markers that can be scaled across the nation.

Maternal obesity rates are disproportionately higher in Low-Middle income countries (LMICs) as India and can have adverse health outcomes for mothers and children. In India, Chopra et al. analyzed the National Family and Health Survey-4 (NFHS-4) data and reported obesity among 12 % of pregnant women 20 years and above, with as high as above 40 % high in over 30 districts in multiple states [8]. Similarly, in children of South Asia, the overall prevalence of overweight and obese children was reported as 1.91 and 0.89 %, respectively [9]. Further, the NFHS-5 survey also found a rise in obesity among children under five years of age in 20 of the 22 states where the study was conducted [10]. A study in rural Haryana also reported the prevalence of macrosomia among live births as 1.3 % (*n* = 12) [11].

Lack of clear recommendations adds to the complexities of screening during pregnancy and infancy. First, there is no standard definition of what constitutes obesity in pregnancy and at birth. The available recommendations are mostly for pre-pregnancy measures [12]. Second, there are ambiguities in the methods for screening obesity, with some using birth weight while others suggesting BMI z-scores or weight-for-length (WFL). The reliability of anthropometric markers in estimating obesity is a substantial challenge. For example, poor sensitivity (47.7 %) and positive predictive value (67.7 %) are noted for BMI [13]. Studies across different settings have shown that high MUAC has high diagnostic accuracy (sensitivity, specificity, and predictive values) for the identification of adiposity (as measured by body composition techniques [14]. Fourth, it is difficult to ensure that trained staff is available to maintain homogeneity and internal validity [13]. Finally, when measured using standard methods, there are high chances of measurement error, often depending on the number of observers, skill and staff turnover [15].

There are several advanced methods with higher reliability for measuring obesity. These include bioelectrical impedance analysis (BIA), deuterium dilution, dual-energy x-ray absorptiometry (DXA), hydrostatic weighing, ultrasound, and magnetic resonance imaging (MRI). Unfortunately, using these instruments is either costly, challenging to implement at the population level, and also requires considerable expertise [16, 17]. Due to these complexities in measuring the total body fat, measuring the thickness of two layers of subcutaneous fat pinched using calipers referred to as total skinfold thickness is generally employed in community settings [18].

It is essential to screen obesity in public facilities using appropriate but realistic methods to assess total body fat in the body. Hence, using total skinfolds for assessing body composition is a quick, convenient, relatively inexpensive method across all ages. However, this requires rigorous training and expertise. In addition to the possibility of high Intra- and inter-observer variability in using the calipers [19], multiple readings in at least three sites are necessary to obtain reliable skinfold thickness. This will not be possible in most public facilities, which are otherwise understaffed, overcrowded, and offer no privacy. It is difficult to ensure frontline health workers have the necessary training and reduce intra- and inter-observer variability in millions of health workers. Therefore, we aimed to assess the validity and determine appropriate cut-off levels of several anthropometric markers as alternatives for total skinfolds in pregnant women and newborn infants in a prospective cohort study.

## Methods

### Study design and subjects

Maternal Antecedents of Adiposity Studying the Transgenerational role of Hyperglycemia and Insulin (MAASTHI) is a prospective pregnancy cohort. A detailed protocol and methods are published elsewhere [20]. In brief, we recruited voluntarily consenting eligible pregnant women from public facilities in Bengaluru, Karnataka, from 2016 to 2019. We excluded participants with Diabetes, Human immunodeficiency virus (HIV), and Hepatitis or their inability to complete the oral glucose tolerance test (OGTT). The included women were aged 18–45 years, having singleton pregnancy before the gestational age of 36 weeks. This was done with the clinical information and reference standards that were available to the performers of the index test and the reference test. We collected the data and measured anthropometry from the voluntarily consenting pregnant women between 14 and 36 weeks. Women were invited for laboratory tests (glucose and haemoglobin) between 24 and 36 weeks. Follow-up was conducted in the women who completed the lab tests, and we considered infants from birth to five months of age.

### Anthropometric measurements

#### Pregnancy

Standing height and weight were measured using the portable stadiometer (SECA 213) and digital weighing scale (Tanita). We recorded weight to the nearest 100 gram with minimal clothing and barefoot. The height was read to the nearest 0.1 cm. Mid-upper arm circumference (MUAC) was measured for the left arm using circumference tape (Chasmors WM02). The women were asked to sit/stand with their back to the measurer, and the elbow flexed at about 90 degrees. The tip of the acromion (the point of the shoulder) and the olecranon processes were palpated and marked with a skin pencil. The distance between these two points was measured by a flexible measuring tape, and a point midway between these two processes was marked on the skin. This midpoint marked the vertical level at which the circumference was measured with the arm hanging by the side. The measuring tape was placed around the upper arm such that the tape was horizontal to the surface. It was ensured that the tape rested firmly against the skin but not pulled too tight to cause indentation of the skin surface [21]. Two readings for each anthropometric measurement were recorded. Head Circumference (HeadC) was measured using Chasmors WM02.

#### Newborn anthropometry

For weight measurement, newborns were placed naked on the digital weighing scale (SECA 354), and two readings to the nearest 0.5 g were taken. The newborn length was measured using Infantometer (SECA 417).

#### Total skinfold thickness

We measured triceps, biceps, and subscapular skinfold thickness in pregnant women between 14 and 36 weeks of pregnancy. For newborns, measurements were done between birth and five months of age. The measurement was conducted on the left side using Holtain Calliper (Holtain, U.K 610ND). Triceps skinfolds were measured over the posterior belly of the triceps muscle of the left arm, halfway between the acromion and the olecranon, on a line passing upwards from the olecranon in the axis of the limb, with the arm extended. Biceps skinfold is measured in the anterior midline of the arm over the biceps on the same level as the triceps skinfold. Subscapular skinfold was measured immediately below the angle of the left scapula, with the arm held by the side of the body. Measurements were made on the left side of the body, and readings were taken 5 s after applying the caliper’s jaws. Three readings to the nearest 0.2 mm were taken.

### Quality control and calibration

All research assistants were trained at the St. Johns Research Institute, Bengaluru, for anthropometric measurements as part of their induction. Competencies of research assistants were assessed at the outset, followed by mandatory annual certification. The relative intra-observer technical error of measurement was below 1.5 % for all measurements and the relative inter-observer technical error of measurement (TEM) was below 2 %. Calibration of all the equipment was done every month.

### Statistical analysis

Descriptive statistics, mean and stratum-wise proportions (as applicable) were generated for socio-demographic and anthropometric characteristics of the study participants. For the anthropometric measures for which multiple readings were available, the arithmetic mean of the non-missing values was used in the analysis. Total skinfold thickness was calculated by summing up the values for biceps, triceps, and subscapular skinfold thicknesses. Curve estimation was done to assess the linearity of the association between total skinfold and other explanatory anthropometric measures (*Transreg* procedure in SAS that utilized the Box-Cox transformation of the dependent variable). The strength of linear association (and statistical significance) was described using simple linear regression. After establishing a linear association between total skinfold and the rest of the anthropometric parameters, Pearson’s correlation analysis between these parameters was assessed. Percentile distribution of maternal skinfold thickness was derived, and based on 90th percentile cut-off, the participating pregnant women were categorized into *high* (above 90th percentile and *normal* skinfold (up to 90th percentile) groups.

For the newborns, the 85th percentile cut-off was used [22]. Receiver operating characteristics (ROC) curve analysis was performed, and separate ROC curves of maternal body weight, head circumference, MUAC, and body mass index (BMI) on 90th percentile cut-off of total skinfold were generated. The optimal cut-off point for each of these measures that corresponded to 90th percentile total skinfold cut-off was determined using the following three methods – (1) Youden’s *J* statistic; (2) minimized distance to (0, 1) point in the ROC curve; and (3) sensitivity-specificity equality [23–27]. In case conflicting cut-off values were obtained from each of the three methods, the results generated by Youden’s *J* statistic procedure were persisted with. For the newborns, the same process was repeated to determine the optimal cut-off of different anthropometric measures corresponding to the 85th percentile cut-off for total skinfolds. Besides the anthropometric parameters used for pregnant women, chest (CC), waist (WC), and hip circumferences (HC) were additional parameters evaluated for newborns. Further, the predictive accuracy of the cut-off points for different anthropometric parameters was evaluated by calculating the proportion of misclassification that would result from the use of determined cut-offs. We also assessed Cohen’s *Kappa* statistic to determine the agreement between the determined cut-off and standard 90th /85th percentile cut-offs for total skinfolds. SAS version 9.4 was used for statistical analyses.

## Results

The research team approached 5725 pregnant women, 913 were ineligible A total of 4812 respondents were recruited, (Supplementary Fig. 1) anthropometry was recorded in 3719 pregnant women, and as seen in Table 1: the mean age was 24.2 years. Majority of them had attained middle school education (91.2 %), 22.7 % had parents with diabetes, 45.1 % of them were primiparous, and one in nine (11.1 %) women were diagnosed with Gestational Diabetes Mellitus (GDM) during the current pregnancy. Of the 3719 pregnant women, 2962 completed the lab tests, women who did not complete the lab tests were not followed up as per the study protocol. Among 2962 pregnant women, there were 60 cases of child death, 290 women had not delivered as of the analysis date, and there were 180 cases lost to follow up as they were not available on phone or at the given address. Infant anthropometry was measured in 2432 infants. The median birth weight was 2.9(0.74), and the interquartile range (IQR) was 0.73(2.6,3.33) kg, and the total skinfold thickness was mm. The median gestational age at delivery was 12.16 weeks and the interquartile range is 2.27. The characteristics of the study population are summarized in Table 1.

**Table 1:** Demographic characteristics of participants (*N* = 3719)

Characteristics	n(%)
***Maternal characteristics (n = 3720)***	
**Maternal age in years (Mean ± SD)**	24.26 ± 4.08
**Gestational age at recruitment in weeks (Mean ± SD)**	23.5 ± 6.01
**Education**	
Primary School and below	337(9.00 %)
Middle School and above	3382 (91.00 %)
**Gravida**	
Primigravida	1467(39.40 %)
Multigravida	2252(60.60 %)
**Parity**	
Nulliparous	1667(44.80 %)
Primiparous	1677(45.10 %)
Multiparous	375(10.10 %)
**Current gestational diabetes status during the assessment**	
Yes	417(11.10 %)
No	3302(88.80 %)
**Anthropometry measurements**	
Weight (kg) (Mean ± SD)	58.95 ± 11.72
Height (cm) (Mean ± SD)	153.9 ± 5.72
Mid-upper arm circumference(cm) (Mean ± SD)	26.0 ± 3.87
Biceps skinfold thickness (mm) (Mean ± SD)	10.63 ± 4.90
Triceps skinfold thickness (mm) (Mean ± SD)	19.56 ± 5.98
Subscapular skinfold thickness (mm) (Mean ± SD)	17.26 ± 6.00
Sum of skinfold thickness (mm) (Mean ± SD)	47.45 ± 14.8
**Delivery outcomes (***n*** = 2432)**	
**Gestational age at delivery in weeks (Mean ± SD)**	38.6 ± 1.6
**Delivery type**	
Vaginal delivery	1341(55.10 %)
Caesarean delivery	1092(44.90 %)
***Infant characteristics (n = 2432)***	
**Sex**	
Male	1257(51.70 %)
Female	1175(48.30 %)
**Age at assessment in days (Mean ± SD)**	12.1 ± 19.3
**Anthropometry measurements**	
Weight (kg) (Mean ± SD)	3.07 ± 0.736
Length (cm) (Mean ± SD)	49.55 ± 3.88
Crown-rump length (cm) (Mean ± SD)	32.59 ± 3.21
Head circumference (cm) (Mean ± SD)	33.76 ± 2.16
Chest circumference (cm) (Mean ± SD)	32.33 ± 2.81
Waist circumference (cm) (Mean ± SD)	30.75 ± 3.72
Hip circumference (cm) (Mean ± SD)	29.16 ± 3.72
Mid-upper arm circumference(cm) (Mean ± SD)	9.89 ± 1.22
Biceps skinfold thickness (mm) (Mean ± SD)	4.05 ± 1.17
Triceps skinfold thickness (mm) (Mean ± SD)	5.19 ± 1.48
Subscapular skinfold thickness (mm) (Mean ± SD)	4.97 ± 1.38
Sum of skinfold thickness (mm) (Mean ± SD)	14.20 ± 3.69

SD, standard deviation; *Others: Christian and Jain; #Occupation: Unskilled: labourer, construction labourer, helper, attender; Peon, cleaner, sweeper, Semi-Skilled: Gatekeeper/Security, Asst. Operator, Asst. electrician, waiter, Skilled: Tailor, carpenter, Driver, plumber, electrician

The optimum cut-off values for the various anthropometric measurements corresponding to the 90th percentile cut-off of the total maternal skinfold thickness are shown in Table 2. Although the cut-off values obtained via different ROC curve methods were not identical, they approximated each other. Given the discordance, the cut-offs generated using Youden’s *J* statistic method − 66.89 kg for weight, 53.39 cm for HeadC, 29.20 cm while considering MUAC, and 27.82 kg/m^2^ for BMI – were selected as optimal.

**Table 2:** Cut-offs for different anthropometric measures corresponding to total skinfold thickness cut-off (90th percentile) in pregnant women, by different methods of ROC curve analysis. [*N* = 3719]

Anthropometric measure	Cut-off corresponding to 90th percentile of total skinfold thickness from different methods		
	Youden’s *J* statistic**	Minimized distance to (0, 1) point in the ROC curve	Sensitivity-specificity equality
Bodyweight (kg)	66.89	66.89	64.94
Head circumference (cm)	53.39	53.39	53.09
Mid-upper arm circumference (cm)	29.2	28.5	28.3
BMI (kg/m^2^)	27.82	27.37	27.49

^*Sum of Biceps, Triceps and Sub−scapular skinfold thickness^

^** In case of discrepancy between cut−offs determined by different methods, the cut−off obtained via Youden’s *J* statistic was considered as standard^

**Fig. 1:**
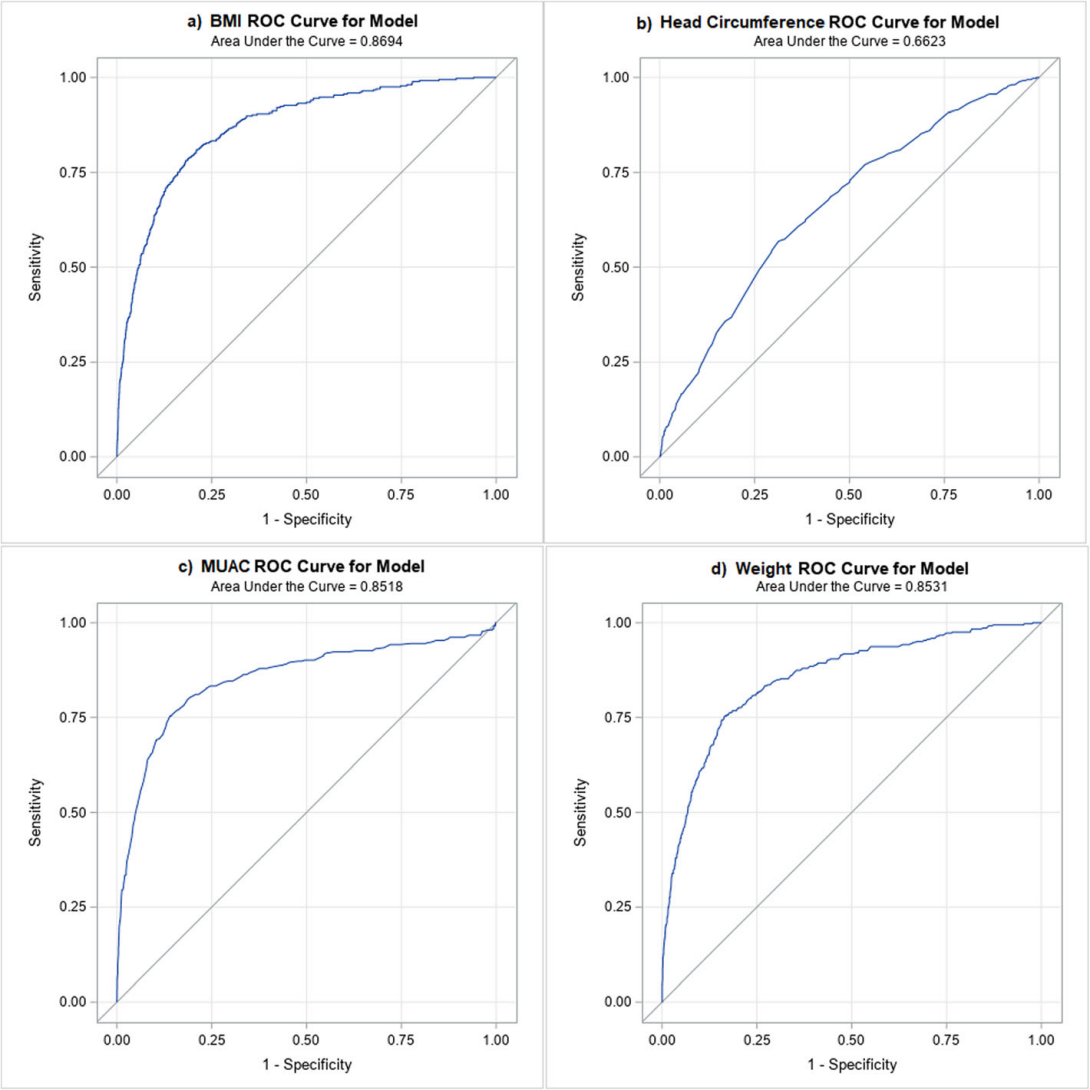
Receiver operating characteristics (ROC) curves of maternal: (**A**) body mass index (BMI), (**B**) head circumference, (**C**) MUAC, and (**D**) body weight

Table 3 depicts the resultant distribution of the 90th percentile of total skinfold according to the optimal cut-off values for the four maternal anthropometric measures, derived from the ROC curve analyses. MUAC cut-off had the least amount of misclassification (15 %), while HeadC cut-off had the highest (worst) misclassification (32.46 %). The highest amount of agreement (as per *Kappa* statistic) with total skinfold was also attributed to MUAC cut-off value (0.42 (95 % CI 0.38–0.46)). The area under the curve was the largest for the BMI cut-off (86.9 %), however, MUAC cut-off was quite close at 85.2 % (Fig. 1). Therefore, considering all aspects, MUAC cut-off emerged as the best possible substitute for measuring total skinfolds in pregnant women. All four anthropometric measures demonstrated statistically significant Pearson’s correlation and slope of linear association with the total skinfold thickness (Supplementary Fig. 2).

**Table 3:** The magnitude of agreement and the extent of misclassification on using different anthropometric measures instead of total skinfold thickness for measurement of body fat in pregnant women (90th percentile cut off). [*N* = 3719]

Anthropometric measure	Cut-off ^#^	Total skin fold thickness percentile		Total misclassification (%)	Kappa coefficient (95 % CI)
		< 90th percentile	> 90th percentile		
Body weight (kg)	< 66.89	2799 (75.26)	90 (2.42)	17.32	0.38* (0.34–0.41)
	≥ 66.89	554 (14.90)	276 (7.42)		
Head circumference (cm)	< 53.39	2304 (61.95)	158 (4.25)	32.46	0.12** (0.1–0.15)
	≥ 53.39	1049 (28.21)	208 (5.59)		
Mid-upper Arm Circumference (cm)	< 29.20	2885 (77.60)	90 (2.42)	15.01	0.42*** (0.38–0.46)
	≥ 29.20	468 (12.59)	275 (7.40)		
BMI (kg/m^2^)	< 27.82	2746 (73.84)	81 (2.18)	18.5	0.36* (0.33–0.40)
	≥ 27.82	607 (16.32)	285 (7.66)		

^**#**Cut off corresponding to 90th percentile of total skinfold thickness^

^*Fair agreement; **Slight agreement; ***Moderate agreement *[Landis & Koch (1977)]*^

**Fig. 2:**
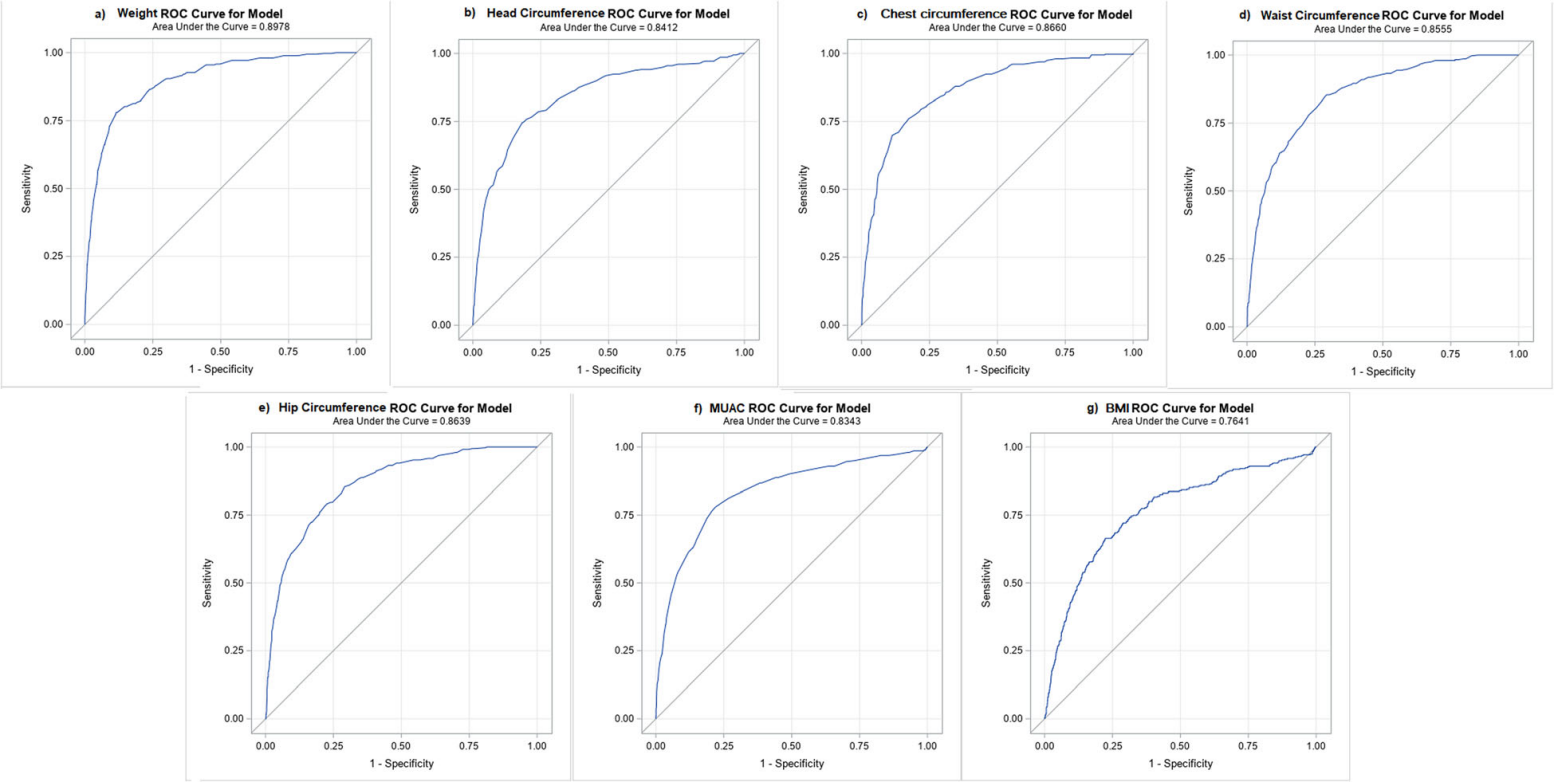
Receiver operating characteristics (ROC) curves of infant anthropometric markers: (**A**) birthweight, (**B**) head circumference, (**C**) chest circumference, (**D**) waist circumference, (**E**) hip circumference, (**F**) MUAC, (**G**) BMI

The optimum cut-off values for the various anthropometric measurements corresponding to the 85th percentile cut-off of the total skinfold thickness of the newborns are shown in Table 4. Similar to the mothers, the cut-off values for each of the seven anthropometric measures for the newborns were not identical but they approximated each other. The Youden’s *J* statistic method revealed the following cut-off values − 3.45 kg for body weight, 35 cm for HeadC, 33.7 cm for CC, 31.7 cm for WC, 30.3 cm for HC, 10.30 cm for MUAC and 13.22 (kg/m^2^) for BMI (Table 4). The Pearson’s correlation and the strength of linear association between the seven anthropometric measures and the total skinfold thickness of the newborns are presented in Supplementary Fig. 3. Each of the seven anthropometric parameters in the newborns showed a statistically significant positive Pearson’s correlation with the total skinfold thickness and had a significantly upward slope in linear regression. On ROC curve analysis, the birth weight of the newborn had the highest area under the curve (89.8 %) among all seven anthropometric measures (Fig. 2).

**Table 4:** Cut-offs for different anthropometric measures that correspond to total skinfold thickness cut-off (85th percentile) for children at birth - determined by different methods of ROC curve analysis. [*N* = 2432]

Anthropometric measure	Cut-off corresponding to 85th percentile of total skinfold thickness from different methods		
	Youden’s *J* statistic**	Minimized distance to (0, 1) point in the ROC curve	Sensitivity-specificity equality
Bodyweight (kg)	3.45	3.40	3.26
Head circumference (Cm)	35.00	34.70	34.60
Chest circumference (Cm)	33.70	33.70	33.40
Waist circumference (Cm)	31.70	32.40	32.40
Hip circumference (Cm)	30.30	30.30	30.50
Mid-upper arm circumference (Cm)	10.30	10.30	10.30
BMI (kg/m^2^)	13.22	12.87	12.88

^*Sum of Biceps, Triceps and Sub−scapular skinfold thickness^

^** In case of discrepancy between cut−offs determined by different methods, the cut−off obtained via Youden’s *J* statistic was considered as standard^

We found that the birth weight cut-off was a good substitute for the total skinfold thickness of the newborns as it demonstrated the lowest amount of misclassification among all seven anthropometric measures (Table 5). The birth weight cut-off also had the highest *Kappa* statistic (0.57) demonstrating a better agreement with 85th percentile of the total skinfold thickness compared to the rest of the anthropometric measures.

**Table 5:** The magnitude of agreement and the extent of misclassification on using different anthropometric measures instead of total skinfold thickness for measurement of body fat among children at birth (85th percentile cut-off). [*N* = 2432]

Anthropometric measure	Cut-off^#^	Total skin fold thickness percentile		Total misclassification (%)	Kappa coefficient (95 % CI)
		≤ 85th percentile	> 85th percentile		
Birth weight (kg) [*N* = 2432]	< 3.45	1839 (75.62)	78 (3.21)	13.00	0.57* (0.52–0.60)
	≥ 3.45	238 (9.79)	277 (11.39)		
Head circumference (cm) [*N* = 2432]	< 35.00	1699 (69.86)	90 (3.70)	19.24	0.42* (0.38–0.46)
	≥ 35.00	378 (15.54)	265 (10.90)		
Chest circumference (cm) [*N* = 2432]	< 33.70	1718 (70.64)	85 (3.50)	18.26	0.45* (0.40–0.49)
	≥ 33.70	359 (14.76)	270 (11.10)		
Waist circumference (cm)	< 31.70	1469 (60.40)	52 (2.14)	27.14	0.34** (0.31–0.37)
	≥ 31.70	608 (25.00)	303 (12.46)		
Hip circumference (cm)	< 30.30	1609 (66.16)	74 (3.04)	22.28	0.39** (0.35–0.43)
	≥ 30.30	468 (19.24)	281 (79.15)		
Mid Upper Arm Circumference (Cm)	< 10.30	1621 (66.65)	78 (3.21)	21.96	0.39** (0.35–0.43)
	≥ 10.30	456 (18.75)	277 (11.39)		
BMI (kg/m2)	< 13.22	1609 (66.19)	119 (4.90)	24.11	0.31** (0.27–0.35)
	≥ 13.22	467 (19.21)	236 (9.71)		

^**#**Cut off corresponding to 85th percentile of total skinfold thickness^

^*Moderate agreement; **Fair agreement *[Landis & Koch (1977)]*^

The extent of misclassification that would result from the use of the newly defined cut-offs instead of the accepted standard, i.e. 85th percentile of total skinfold in newborns, along with the amount of agreement (expressed by *Kappa* statistic) between each of the seven measures and total skinfolds cut-off. Our results indicate that the birth weight cut-off (3.45 kg) had the least amount of misclassification (13.00 %) against the 85th percentile of the total skinfold thickness, while the BMI cut-off had the highest (worst) misclassification (24.11 %). The highest value of *Kappa* statistic was also attributed to Birth weight (0.57 (0.52–0.60)] followed by the head and chest circumferences, respectively. The cut-offs for circumferences at the waist, hip, and mid-upper arm and BMI showed *fair* agreement with total skinfold thickness. Therefore, on the basis of the extent of misclassification and agreement, the birth weight cut-off emerged as the best substitute (among all anthropometric measures).

## Discussion

There is a need to use feasible and accurate nutritional status indicators in pregnant women and newborn children to identify adiposity, an independent cardiometabolic risk factor. The burgeoning epidemic of obesity impacts all age groups and negatively impacts the life course and generations. Our results indicate that MUAC higher than 29.2 cm can serve as a suitable alternative to total skinfolds-based assessments for obesity screening in pregnancy in resource-constrained public health facilities. Similarly, a birth weight cut-off of 3.45 kg can be considered for classifying obesity among newborns.

Pre-pregnancy measurements are rarely available in most Indian setting [28]. As per the national survey [29], 59 % rural and 41 % of urban pregnant women avail public facilities for antenatal care, they mostly have their first antenatal visit late in the first trimester (or even later), making the bodyweight an unreliable indicator for assessment of overweight or obesity in pregnancy [30]. Since body weight is also integral to BMI estimation, this also suffers from the same limitation as a marker for obesity. Therefore, to obtain a reliable marker for obesity at any given point during the gestational period, we attempted to use MUAC measurements. Our results showed concordance with the studies from Sri Lanka and Nigeria, wherein the reliability of inexpensive MUAC is validated [31, 32]. Measuring MUAC in pregnancy eliminates the need for sophisticated equipment and calculations and is a reliable proxy of pre-pregnancy body fat and nutrition. These reasons also make MUAC a popular and feasible choice in public facilities [33–35]. A recent study conducted among adolescents, lactating, and parous non-pregnant women in one of the most impoverished regions in India reported that MUAC could be a viable marker for assessing women’s nutritional status in community settings [27]. Maternity care guidelines in South Africa, a country with a similar economic profile and maternal health challenges as India, recommend using MUAC greater than 33 cm to indicate obesity in pregnant women [34]. The evidence from Argentina suggests MUAC cut-off points according to the gestational age [35].

Birth weight is a reliable predictor of body composition in newborns, explaining up to 84 % of body fat in newborns [36, 37]. Previous studies have shown that Indian babies preserve more subscapular skinfold thickness at birth even though these children had a lower birth weight [38]. However, this was not replicated in recent studies that showed that Skinfold thicknesses in Indian babies were similar to those reported in a Western population with comparable birth weights. Some of these studies used more accurate measurements of body composition like deuterium dilution and air displacement plethysmograph [37, 39, 40]. Studies have shown a significant positive correlation between body weight and body fate percentage across the weight range of 2.3–4 kg [40]. The available evidence supports our finding that intrauterine growth is best assessed by weight at birth [41, 42]. Similar findings were also found in other LMICs [36, 43].

In India, measuring MUAC in pregnancy and birth weight to assess obesity can help plan and prevent potential adverse outcomes. We recently showed that maternal obesity is an independent risk factor for neonatal adiposity [44]. Total skinfold measurement, the preferred method for assessing obesity, is often impeded by the dearth of trained staff, time, and costly equipment. In comparison, MUAC and birth weight measurements can be incorporated relatively easily in antenatal care services for immediate use in all hospitals. The weighing scales are available in all labour rooms, including rural health centres. Therefore, the measurement of birth weight can be done immediately after birth. This can be further validated in other geographies and settings (such as private hospitals) to arrive at a national consensus for cut-off so that appropriate obesity control measures can be taken in early childhood to prevent the deleterious health consequences in their adult life. Both the anthropometric markers as alternatives for skinfold thickness in our study demonstrated the feasibility for use in public facilities due to the usability and costs involved.

Firstly, some of the limitations are the need to ensure adequate training for the healthcare staff for MUAC measurement. However, MUAC is less resource and skill-intensive compared to skinfold thickness assessment. Secondly, there could be misclassification resulting from using a substitute measure for total skinfold thickness in obesity measurement; a certain proportion of the population may wrongly get classified obese (or vice versa) when they are not so. Further validation studies in India can establish the reliability and validity to steer policy-level actions to prioritise screening obesity in pregnancy. The third limitation is that this study mostly represents the source population comprising low-middle-income women who attend public facilities in Bengaluru. This needs to be validated in an even larger population to prove its wider applicability. However, we were able to capture the measurements among a large sample size of mother-child dyads and thus have shown the use at public facilities for urban populations that can be applied across the country.

## Conclusion

Mid-upper arm circumference and birth weight can be used as markers of skinfold thickness, reflecting total body fat in pregnant women and infants, respectively. These two anthropometric measurements could substitute for skinfold thickness in low- and middle-income urban India settings. Our results suggest that the MUAC and birth weight be used in pregnant women and infants, respectively, as markers for effective screening tools for detection of obesity in Indian states and other similar settings. The simple technique and low costs associated with measurements can enable the implementation by frontline health workers including in rural areas.

## Supplementary information

**Additional file 1**

**Additional file 2**

**Additional file 3**

**Additional file 4**

## Data Availability

The data that support the findings of this study are not publicly available, but applications for data access can be submitted to the corresponding author on reasonable request.
