# Supplementary figures and images for: Mid-upper arm circumference in pregnant women and birth weight in newborns as substitute for skinfold thickness: findings from the MAASTHI cohort study, India
Source: BMC Pregnancy Childbirth. 2021 Jul 6;21:484. doi: 10.1186/s12884-021-03915-1 (PMC8258932; doi:10.1186/s12884-021-03915-1)

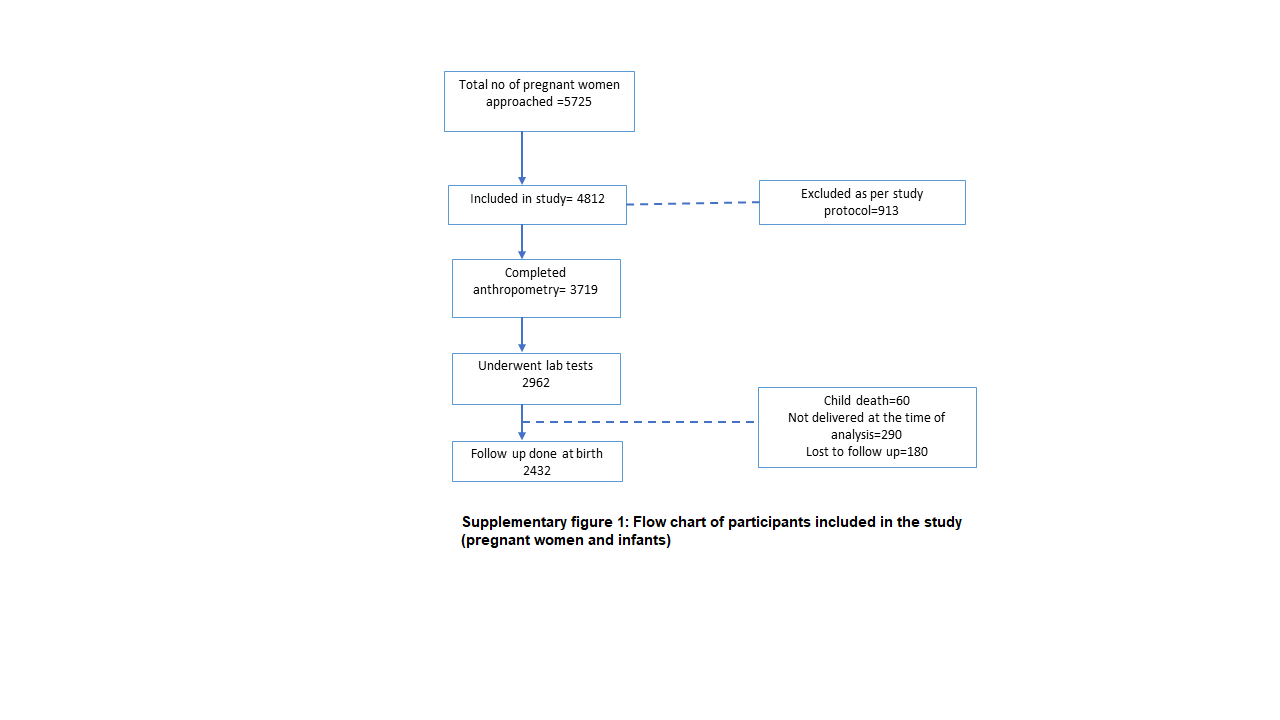

Supplement: Supplementary file 1 — Additional file 1 [file 12884_2021_3915_MOESM1_ESM.png]

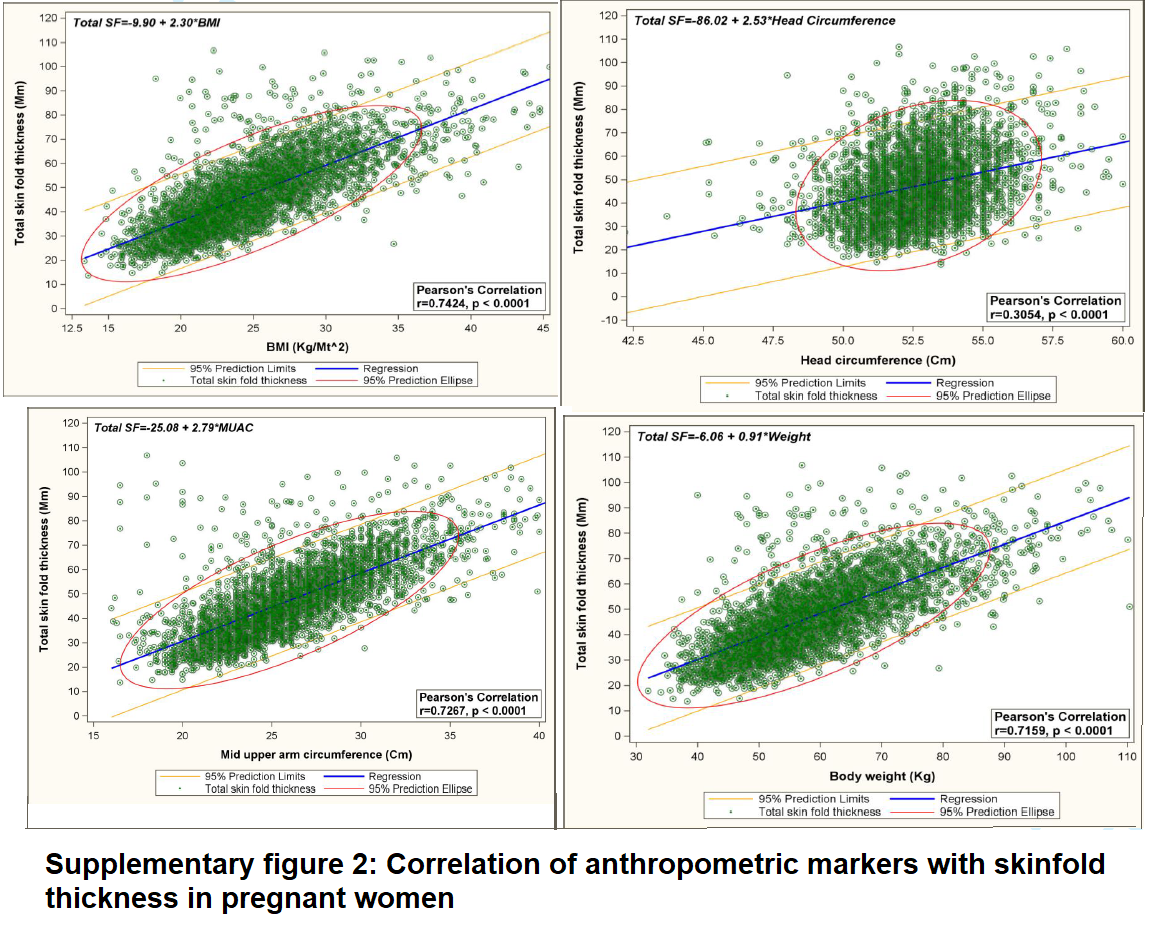

Supplement: Supplementary file 2 — Additional file 2 [file 12884_2021_3915_MOESM2_ESM.png]

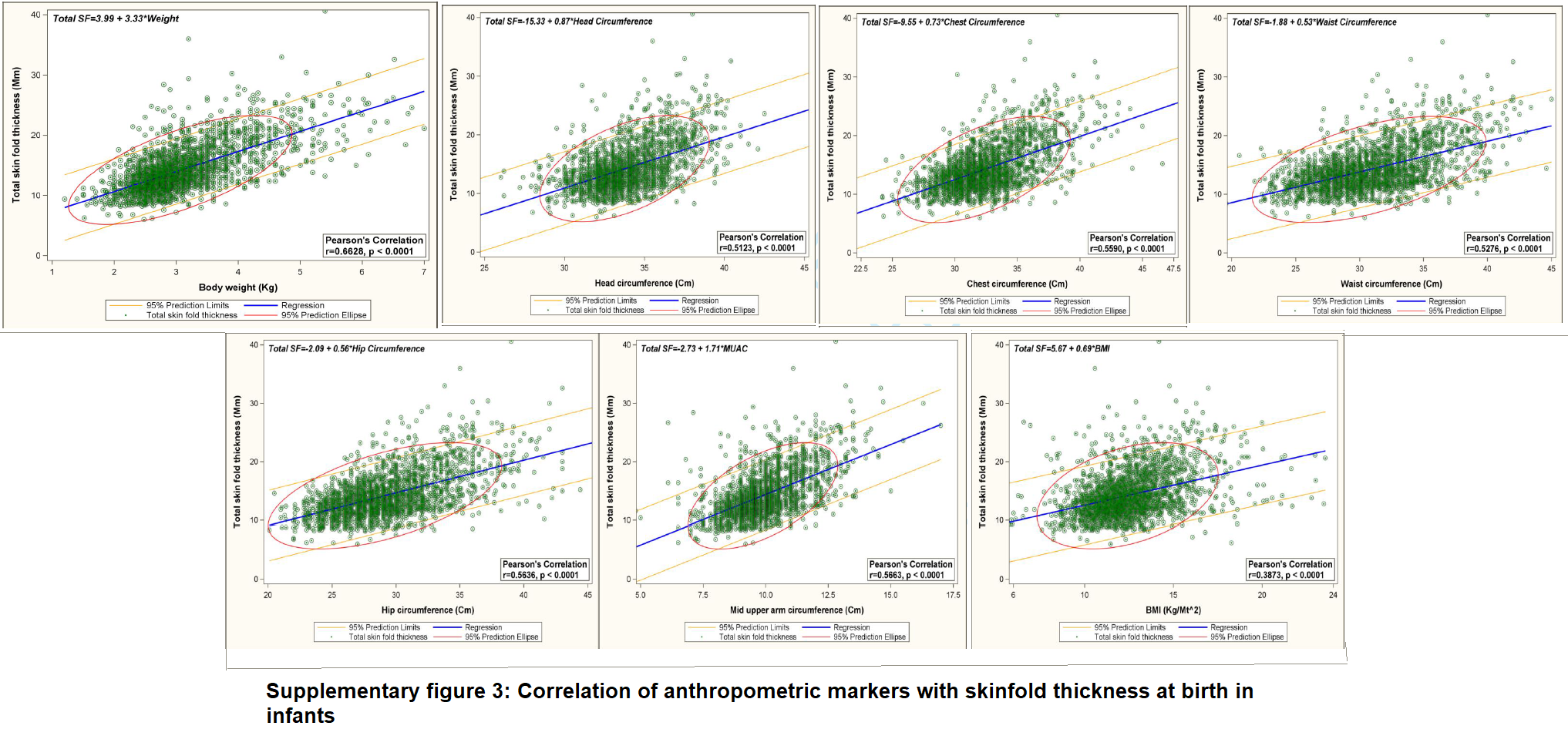

Supplement: Supplementary file 3 — Additional file 3 [file 12884_2021_3915_MOESM3_ESM.png]
