# Supplementary material for: Mid-upper arm circumference in pregnant women and birth weight in newborns as substitute for skinfold thickness: findings from the MAASTHI cohort study, India
Source: BMC Pregnancy Childbirth. 2021 Jul 6;21:484. doi: 10.1186/s12884-021-03915-1 (PMC8258932; doi:10.1186/s12884-021-03915-1)
Supplement: Supplementary file 4 — Additional file 4 [file 12884_2021_3915_MOESM4_ESM.docx]

| **Supplementary table 1. Statistical properties of the cut-off corresponding to 90th percentile of total skin fold thickness in pregnant women using different methods. [N=3719]** | | | | | | | | |
| --- | --- | --- | --- | --- | --- | --- | --- | --- |
| **Anthropometric measure** | **Cut-off point*** | **Area under the curve (%)** | **Youden's *J* statistic for the cut-off** | **Sensitivity corresponding to cut-off (%)** | **Specificity corresponding to cut-off (%)** | **Positive predictive value corresponding to cut-off (%)** | **Negative predictive value corresponding to cut-off (%)** | **% of pregnant women classified as having high body fat** based on the cut-off of anthropometric measure** |
| Body weight (Kg) | 66.89 | 85.31 | 0.5889 | 75.41 | 83.48 | 33.25 | 96.89 | 22.32 |
| Head circumference (Cm) | 53.39 | 66.23 | 0.2555 | 56.83 | 68.72 | 16.55 | 93.58 | 33.8 |
| Mid-upper arm circumference (Cm) | 29.2 | 85.18 | 0.6139 | 75.34 | 86.04 | 37.01 | 96.98 | 19.98 |
| BMI (Kg/Mt^2^) | 27.82 | 86.94 | 0.6001 | 78.14 | 81.87 | 31.99 | 97.17 | 24.04 |
| *Cut-off based on Youden’s *J* statistic | | | | | | | | |
| **Corresponding to 90th percentile of total skin-fold thickness | | | | | | | | |

| **Supplementary table 2. Statistical properties of the cut-off corresponding to 85th percentile of total skin fold thickness in children at birth using different methods. [N=2432]** | | | | | | | | |
| --- | --- | --- | --- | --- | --- | --- | --- | --- |
| **Anthropometric measure** | **Cut-off point*** | **Area under the curve (%)** | **Youden's *J* statistic for the cut-off** | **Sensitivity corresponding to cut-off (%)** | **Specificity corresponding to cut-off (%)** | **Positive predictive value corresponding to cut-off (%)** | **Negative predictive value corresponding to cut-off (%)** | **% of newborns classified as having high body fat** based on the cut-off of anthropometric measure** |
| Bodyweight (kg) | 3.45 | 89.78 | 0.6657 | 78.03 | 88.54 | 53.79 | 95.93 | 21.18 |
| Head circumference (Cm) | 35.00 | 84.12 | 0.5645 | 74.65 | 81.80 | 41.21 | 94.97 | 26.44 |
| Chest circumference (Cm) | 33.70 | 86.6 | 0.5877 | 76.06 | 82.72 | 42.93 | 95.29 | 25.86 |
| Waist circumference (Cm) | 31.70 | 85.55 | 0.5608 | 85.35 | 70.73 | 33.26 | 96.58 | 37.29 |
| Hip circumference (Cm) | 30.30 | 86.39 | 0.5662 | 79.16 | 77.47 | 37.52 | 95.60 | 32.20 |
| Mid-upper arm circumference (Cm) | 10.30 | 83.43 | 0.5607 | 78.03 | 78.05 | 37.79 | 95.41 | 30.14 |
| BMI (kg/m^2^) | 13.22 | 76.41 | 0.4408 | 66.48 | 77.60 | 33.67 | 93.12 | 28.84 |
| *Cut-off based on Youden’s *J* statistic | | | | | | | | |
| **Corresponding to 85th percentile of total skin-fold thickness | | | | | | | | |
